# Dinosaur Peptides Suggest Mechanisms of Protein Survival

**DOI:** 10.1371/journal.pone.0020381

**Published:** 2011-06-08

**Authors:** James D. San Antonio, Mary H. Schweitzer, Shane T. Jensen, Raghu Kalluri, Michael Buckley, Joseph P. R. O. Orgel

**Affiliations:** 1 Operations, Orthovita, Inc., Malvern, Pennsylvania, United States of America; 2 Department of Marine, Earth and Atmospheric Sciences, North Carolina State University, Raleigh, North Carolina, United States of America; 3 North Carolina Museum of Natural Sciences, Raleigh, North Carolina, United States of America; 4 Museum of the Rockies, Montana State University, Bozeman, Montana, United States of America; 5 Department of Statistics, The Wharton School, University of Pennsylvania, Philadelphia, Pennsylvania, United States of America; 6 Division of Matrix Biology, Department of Medicine, Beth Israel Deaconess Medical Center and Harvard Medical School, Boston, Massachusetts, United States of America; 7 Department of Biological Chemistry and Molecular Pharmacology, Harvard-Massachusetts Institute of Technology Division of Health Sciences and Technology, Boston, Massachusetts, United States of America; 8 Manchester Interdisciplinary Biocentre, Faculty of Life Sciences, The University of Manchester, Manchester, United Kingdom; 9 Department of Archaeology, The University of York, York, United Kingdom; 10 Department of Biology, Pritzker Institute of Biomedical Science and Engineering, Illinois Institute of Technology, Chicago, Illinois, United States of America; University of Cambridge, United Kingdom

## Abstract

Eleven collagen peptide sequences recovered from chemical extracts of dinosaur bones were mapped onto molecular models of the vertebrate collagen fibril derived from extant taxa. The dinosaur peptides localized to fibril regions protected by the close packing of collagen molecules, and contained few acidic amino acids. Four peptides mapped to collagen regions crucial for cell-collagen interactions and tissue development. Dinosaur peptides were not represented in more exposed parts of the collagen fibril or regions mediating intermolecular cross-linking. Thus functionally significant regions of collagen fibrils that are physically shielded within the fibril may be preferentially preserved in fossils. These results show empirically that structure-function relationships at the molecular level could contribute to selective preservation in fossilized vertebrate remains across geological time, suggest a ‘preservation motif’, and bolster current concepts linking collagen structure to biological function. This non-random distribution supports the hypothesis that the peptides are produced by the extinct organisms and suggests a chemical mechanism for survival.

## Introduction

While it is widely accepted that proteins have the potential to survive significantly longer periods of time than DNA [1], persistence of original bone proteins in fossils at least 68 million years old is controversial [2], [3], despite multiple lines of evidence supporting this hypothesis [4], [5], [6], [7], [8], [9]. Current temporal limits for survival of original biomaterials [10], [11] are based upon theoretical kinetics and laboratory experiments designed to simulate protein diagenesis through exposure to harsh conditions (e.g. low pH and high temperature [10], [12]) and predict complete degradation of measurable biomolecules in well under a million years if degradation proceeds at simulated rates. Modeled degradation of DNA [13] places temporal limits of ∼100,000 years (at a constant 10°C), whereas models of protein degradation (e.g. [1], [14]) extend this to a few million years (at a constant 10°C). However, these predictions have been surpassed (e.g. [15]), supporting the suggestion that current models may not be appropriate, in part because they do not consider the molecules in their native state (i.e., folded, closely-packed, cross-linked or, in the case of bone, stabilized by association with the mineral phase [16]). Recovery of what appear to be cells, blood vessels and tissues from multiple fossils from varying ages and depositional settings [4], and protein sequence data from two dinosaurs [5], [6], [7], [9], also suggests that these models may be incomplete. Examining endogenous biomolecules other than DNA avoids synthetic amplification and reduces contamination issues that significantly impeded early ancient DNA research. Technological improvements in recent years, including soft ionization mass spectrometry, allow increased detection of minute traces of biomolecules that may persist for extended periods of time via crystal encapsulation [17], [18], even in the presence of exogenous contamination that precluded earlier forms of analysis such as amino acid composition analyses and stable isotope analyses [13].

The possibility of using information contained in ancient molecules to address contemporary questions of basic biology and ecology is intriguing, and has unexpected potential beyond paleontology. For example, identifying the elements of the collagen fibril most resistant to degradation in fossils may lead to the rational design of collagenous scaffolds with enhanced *in vivo* longevities to support tendon or bone regeneration in humans. Similarly, identifying naturally occurring modifications on these molecules that contribute to preservation may also shed light on molecular-based disease processes. We show here that molecular preservation is linked to protein function, and discuss how sequences of ancient peptides can test models of molecular function in extant organisms. In addition, we show how models of extant protein function suggest a mechanism for the survival of proteins in exceptionally well preserved fossils.

## Results and Discussion

**Figure 1:**
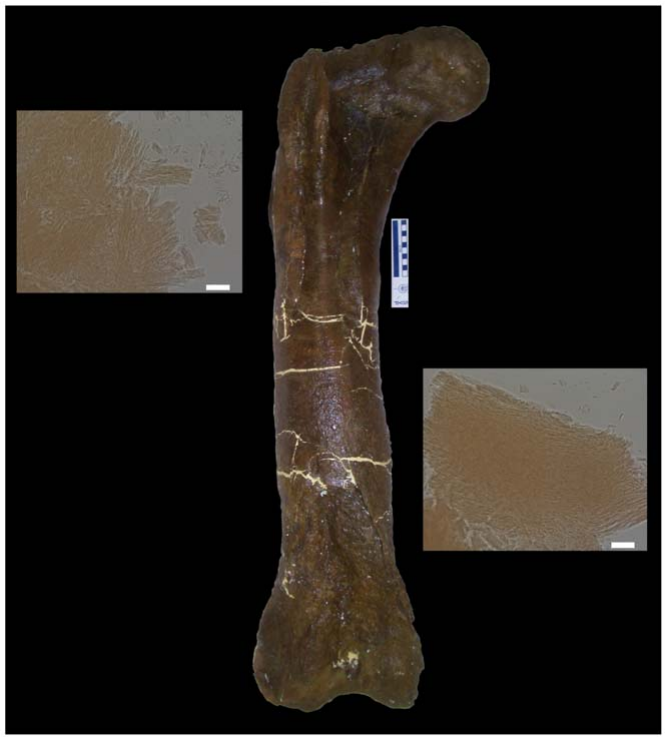
*Tyrannosaurus rex* femur (MOR 1125) from which demineralized matrix (insets; bars, 20 **µ**m) and peptides were obtained. Courtesy Museum of the Rockies.

Type I collagen peptides were extracted and sequenced from ∼ 68 million years old fossils of *Tyrannosaurus rex* (Museum of the Rockies [MOR] 1125) [5], [7], (**Fig. 1**). However, despite multiple lines of evidence to support the presence of collagen, including *in situ* antibody binding, the endogeneity of MOR 1125 peptides was disputed, and the sequences instead were suggested to arise from either microbial invasion [19], extant collagens introduced in laboratory experiments [2], or even statistical artifact [3]. Collagen peptide sequences were subsequently derived from a second dinosaur, *Brachylophosauraus canadensis* (MOR 2598) [9], and included many of the earlier lines of supporting evidence as well as independent replication of data in multiple labs.

**Figure 2:**
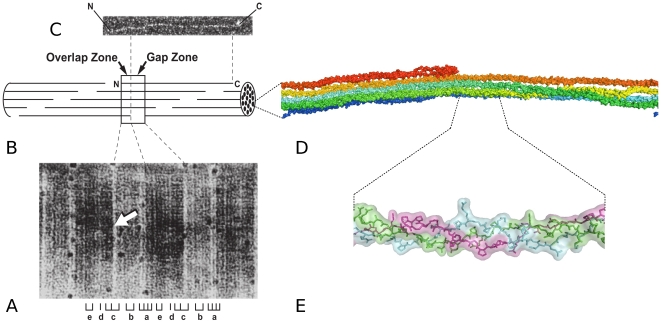
The collagen fibril (A) is composed of triple-helical monomers that polymerize in an overlapping fashion (B), and are derived from proteolysis of the soluble procollagen precursor (C). Fibrils appear as periodic banded structures by electron microscopy; one D-period (expanded two-dimensional view of 67 nm segment of microfibril, box) contains the complete collagen sequence from elements of five monomers and includes an overlap and gap zone; arrow, left border of overlap zone. Image of the X-ray diffraction-derived fibril subunit structure: the microfibril (D) shows aggregates of five triple-helical, rope-like monomers; magnified view shows triple helix containing three peptide chains (two α1 and one α2 chains) (E). Many thousands of microfibrils polymerize and cross-link to form cable-like collagen fibrils of vertebrates. Modified from original research [33].

Surprisingly, advances in collagen biology also support the authenticity of the fossil peptides. The molecular structure of collagen favors preservation. The triple-helical arrangement and intra- and intermolecular cross-links confer stability upon this ubiquitous structural molecule [20], [21], [22], [23], [24], [25]. Additionally, when collagen is surrounded by or adsorbed to mineral surfaces, as in bone, its preservation potential is greatly enhanced (e.g. [18], [26], [27], [28], [29], [30]). In fibrillar collagens, individual triple-helical molecules aggregate, forming a fibril with a characteristic 67 nm banding pattern that is readily recognized by electron microscopy (**Fig. 2**) [31], [32]. Within each 67 nm wide D-period, segments of neighboring molecules are referred to as monomers 1–5 (**Fig. 2**), and specific functional regions have been mapped to each monomer using a variety of experimental approaches [33], [34], [35].

The stability and unique function conferred by the triple-helical structure of collagen has been known for over forty years, but just how molecules assemble into microfibrils to form the massive cable-like fibrils in tissues has been less well understood. However, recent advances in technology have allowed molecular resolution images of type I collagen microfibrils and fibrils [35], [36]. This new information, coupled with non-random distribution of collagen functional sequences and mutations [33], has led to the formation of a testable model linking structure to function in this massive protein assemblage. Discrete cell- and matrix- interaction domains have been identified, and collagen-binding ligands that cooperatively carry out fibril functions have been recognized.

**Figure 3:**
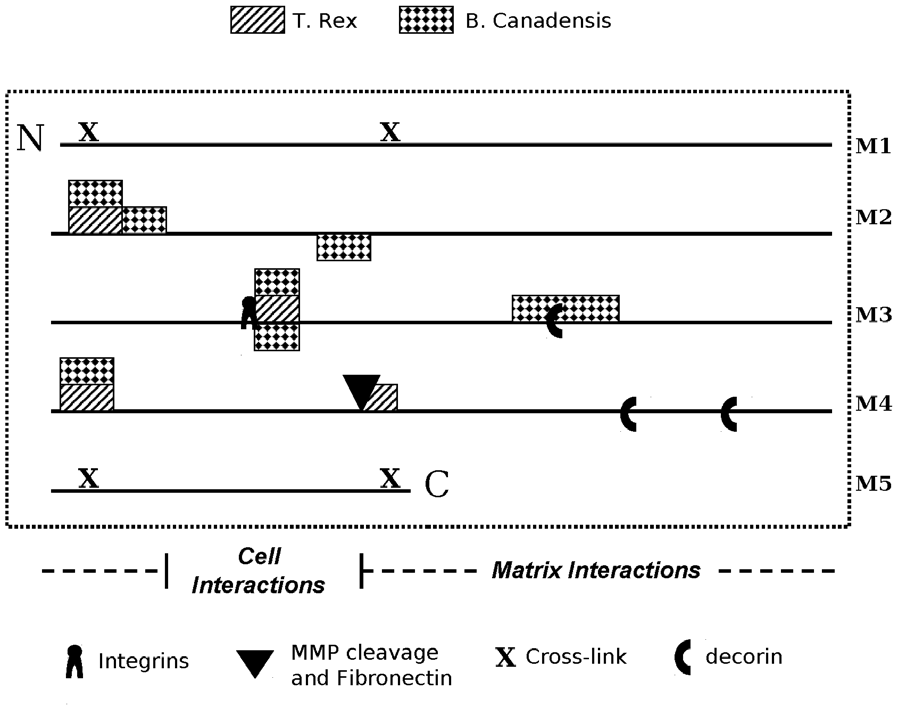
Dinosaur peptide sequence positions were mapped on the two dimensional human collagen fibril D-period schematic^33^.

**Figure 4:**
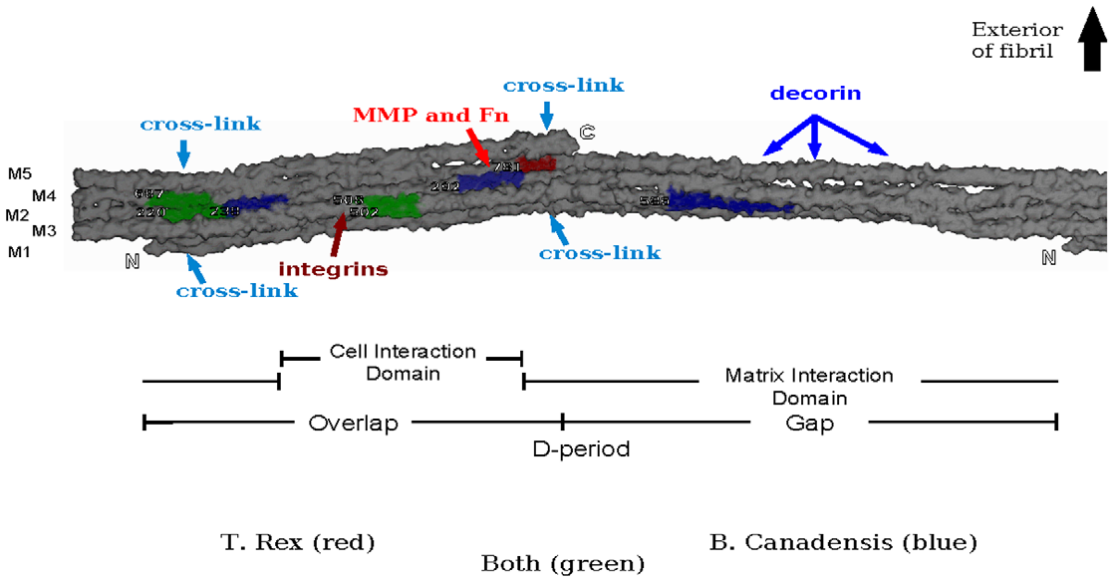
X-ray diffraction model of the rat collagen microfibril *in situ*; Integrins, predominant cell-binding site; MMP, matrix metalloproteinase cleavage site; FN, fibronectin binding site; decoron, decorin proteoglycan core protein binding sites; putative cell and matrix interaction domains^35^.

We reasoned that particular functional molecular regions may contribute to their preferential resistance to biological degradation throughout the lifetime of an individual organism. This property not only needs to remain highly conserved through species but also may render those regions resistant to degradation in the burial environment. Thus, molecular models for differential functions of collagen fibril domains or sequences may provide a chemical or structural rationale for preservation. We mapped eleven fossil-derived peptide sequences from two dinosaurs, *Tyrannosaurus rex* and *Brachylophosauraus canadensis* [7], [9], [37] on molecular models of extant human and rat collagens [33], [34] (**Table 1** **,** **Figs. 3** **and** **4**). These peptides represent eight sequences which localize to seven regions of the monomer, and comprise less than fifteen percent of the length of the collagen triple helix. They were non-randomly distributed in several respects (**Fig. 3** **and Statistical Analyses** [**see** **Materials and Methods**]). In particular, fossil sequences mapped to regions of the protein partly shielded by tight molecular packing (**Fig 4**) [34], which may physically stabilize and protect them from enzymatic degradation, thus contributing to their preservation. Comparing the amino acid compositions of fossil peptides with sequences of the entire human protein for predicted properties such as hydrophobicity, polarity and charge revealed that most fossil peptides were from regions of collagen which contain relatively few acidic residues [38], and eight of the peptides (five sequences) lacked such residues altogether, which would limit their solubility and propensity for proteolytic degradation (**Table 1**). Also, five peptides mapped to a uniquely hydrophobic fibril region [39]. The results imply that the most stable regions of the protein are those with a more hydrophobic, less acidic nature. That the more exposed, charged regions of collagen with high densities of trypsin cleavage sites yielded fewer fossil peptides suggested their susceptibility to proteolysis in early diagenesis, and supports non-random degradation and preservation patterns for the diverse type I collagen sequence set in fossil bone. It is also interesting to note that perhaps the least stable region, the hydroxyproline deficient thermally-labile domain located towards the C-terminal end of the molecule [40], is not represented by any of the fossil peptides.

**Table 1:** Number of Acidic Residues.

Peptide	Species	chain	Amino Acid Sequence	Position	*Observed*	*Predicted*
1	T-Rex	**α1<**	GATGAPGIAGAPGFPGAR	**220–237**	0	2.1
2	T-Rex	**α1<**	GAAGPPGATGFPGAAGR	**687–704**	0	2.0
3	T-Rex	**α1<**	GVQGPPGPQGPR	**508–519**	0	1.4
4	T-Rex	**α1<**	GVVGLPGQR	**781–789**	0	1.0
5	B-Can	**α1<**	GLTGPIGPPGPAGAPG**D**K-G**E**AGPSGPPGPTGAR	**586–618**	2	3.8
6	B-Can	**α1<**	GSAGPPGATGFPGAAGR	**687–704**	0	2.0
7	B-Can	**α1<**	GATGAPGIAGAPGFPGAR	**220–237**	0	2.1
8	B-Can	**α1<**	GVQGPPGPQGPR	**508–519**	0	1.4
9	B-Can	**α1<**	GPSGPQGPSGAPGPK	**238–252**	0	1.7
10	B-Can	**α2**	GSNG**E**PGSAGPPGPAGLR	**292–309**	1	2.0
11	B-Can	**α2**	GLPG**E**SGAVGPAGPPGSR	**502–519**	1	2.0

**Chemical characteristics of fossil peptides.** Dinosaur peptide sequences were obtained from the literature and their alpha chain location and amino acid positions on the human collagen model determined. The prevalence of acidic residues (*bolded, underlined*) in the peptides was lower than predicted for “average” peptides of comparable lengths from pepsinized human collagen [38], implying that regions of collagen with a less acidic nature were preferentially preserved in the fossils.

All fossil-derived peptides mapped to monomers 2, 3, and 4 on the extant collagen models. The remaining monomers, 1 and 5, are joined across microfibrillar layers by intermolecular cross-links that, while stabilizing the molecule and protecting from enzymatic attack, may also hinder peptide extraction. In fact, the only position where alpha 1 chain peptides (Peptides 3 and 8) co-localize with an alpha 2 chain peptide (Peptide 11) mapped to the integrin binding site that promotes cell-collagen interactions, angiogenesis, and osteoblast differentiation; its fibril location and association with severe mutations also suggest its crucial nature [33] and hence strong selective pressure for conservation of sequence. One peptide (Peptide 4) mapped to the Matrix Metalloproteinase-1 (MMP-1) cleavage domain crucial for collagen remodeling, and a site for fibronectin binding. In living tissues, the integrin binding site and MMP-1 cleavage/fibronectin binding sequences are somewhat buried under the surface of the collagen fibril, thus fibril proteolysis or injury may be needed to render them available for cell-collagen interactions and tissue regeneration [35]. The molecularly “sheltered” environment required to protect crucial biological function may also account for enhanced survival of those protein regions in fossils. Although the majority of the dinosaur peptides are from highly conserved regions of the molecule, both of the alpha 2 chain peptides are highly variable [41], [42]. That they are not exclusively from sequences with a high similarity to residues in public databases, suggests that the peptides were not identified solely because they derive from highly conserved sequences; thus, the gaps in our model are not simply due to the lack of peptide identification due to divergence from known organisms. Additional preservation potential may be conferred by association with biomineral, especially if some regions of the collagen molecule are more intimately associated with mineral than others. Conversely, the absence of peptide matches elsewhere in the molecule may be due to lack of response to trypsin resulting from unusual post-mortem modifications which may also confer resistance to proteolytic degradation and contribute to preservation over time [20]. Additional collagen sequences may have survived over time, but because of chemical modification or lack of representation in current databases, may not have been recognized by existing search algorithms and therefore not identified in original analyses.

Our results add to the evidence provided by sequence data [5], [7], [9], [37], molecular phylogenetic analyses [8], [9], microstructure [4], [6], [9], [43] and immunoreactivity to anti-collagen antibodies [6], [9], [43], that supports persistence of elements of native collagen fibril structure across geological time in some fossils. Most of the peptide sequences aligned perpendicularly with one or more other sequences on the fibril model, implying that neighboring triple-helical segments, or fragments thereof, may have been preserved *en bloc.* If supported by further peptide recovery and mapping, this observation would validate current models of collagen monomer arrangement in the fibril [35], [44].

Mapping the distribution of fossil collagen peptides observed using mass spectrometry to models of collagen function demonstrates that preservation of fossil-derived collagen sequences concurs with current concepts of collagen biology, and provides a molecular mechanism for the preservation of this protein in fossil bone. Moreover, these findings support the endogeneous source and longevity of fossil-derived peptides, because peptides arising from recent contamination are expected to be more concentrated and random in distribution. They would not be expected to be over-represented in regions that so well reflect collagen fibril structure/function relationships in native vertebrate tissue [33], [34].

Finally, by showing that functionally crucial protein regions are more stable than others over geologic time, we provide insight into selective pressures constraining the molecular structure, function, and hence sequence, of collagen. Paleoproteomics therefore not only holds significant promise for elucidating evolutionary relationships between extinct and extant organisms, but is potentially useful for enhancing our understanding of protein function in living animals. Also, elucidating molecular functions of extant proteins may help predict proteins or protein regions most likely to preserve in fossils, as has also been shown for the highly-conserved and structurally sheltered mineral-binding mid-region of the bone protein osteocalcin [45]. As technologies continue to improve in both sensitivity and resolution, the recovery of additional protein sequences from fossils will be enhanced. The understanding of preferential preservation driven by molecular function may be used to adapt search algorithms to optimize studies of ancient molecules recovered from multiple extinct taxa. The recovery of additional sequences, allowed by these advances, may shed further light on the biology of extracellular matrix superstructures of living organisms.

## Materials and Methods

### Peptide sequences

Eleven peptides representing eight sequences recovered from the bones of *Tyrannosaurus rex* (MOR 1125) and *Brachylophosauraus canadensis* (MOR 2598 were obtained from previous publications [5], [6], [7], [9].

### Peptide mapping on collagen models

#### Human microfibril

The two dimensional expanded schematic of the human collagen fibril D-period used here was as presented previously [33]. Positions of select binding sites and functional domains from the D-period ligand binding and mutation map [33] are indicated by symbols placed next to the relevant sequences on the schematic, and the positions of dinosaur peptide sequences were mapped to homologous human sequences according to their linear distance from the N-terminus of the collagen triple helix.

#### Rat microfibril

The three dimensional collagen microfibril model used in this study was composed from the packing structure of rat tendon type I collagen molecules *in situ* [35]–[36]. This molecular model was constructed based on the primary sequences of the α1 and α2 chains of rat collagen, and the superhelical parameters were established from crystallographic structure determinations of collagen-like peptides constrained within the lower resolution fiber diffraction molecular envelope [35]. To map the position of the dinosaur peptide sequences on the three-dimensional rat microfibril, solvent-accessible surface calculation and rendering was performed using SPOCK [46] with the default probe size of 0.14 nm to compose a molecular outline. The Cα “worm” traces of relevant portions of individual triple helices were marked (see **Fig. 4** for color key) to indicate the positions of peptide sequences from either *Tyrannosaurus rex* or *Brachylophosauraus canadensis*, or both (where they co-localized on the collagen molecule). The significant homology between vertebrate collagen protein sequences justifies the approach of localizing functional domains of human type I collagen on the rat type I collagen microfibril.

### Statistical Analysis of Peptide Distributions on Collagen

We show the alignment of the eleven dinosaur peptides with homologous sequences on the human collagen map (**Fig. 3**). By visual inspection, the peptide locations appear to be non-random in several ways. For example, there appears to be co-localization between peptides from the two species on the collagen monomer at three positions. The most interesting finding is that at one of these positions, the alpha 1 chain peptide also co-localizes with its matching alpha 2 chain peptide which occurs at the integrin binding site. Also, all peptides map to Monomers 2, 3, and 4, but not to Monomers 1 and 5. We evaluated the statistical significance of these and other seemingly non-random features through their comparison to a null hypothesis of completely random alignment of the peptides to the collagen map. The null distribution of random alignment was calculated via simulation: a large number (m = 100,000) of simulated maps were generated where the eleven peptides were randomly placed. Each map was generated by sampling eleven random numbers from a discrete uniform distribution (with replacement) among all possible map locations. The uniqueness of a given feature of the peptide alignment to the collagen map was evaluated by calculating the proportion of random maps sharing that feature. We refer to this proportion as the randomization p-value, and deem features with an exceedingly small p-value to be significant (i.e. very few random maps share that feature). We calculated the randomization p-value for nine features of the peptide alignment to the human collagen map. In calculating our threshold for declaring significance, we must account for the fact that we are performing multiple tests (for nine different features). We use the conservative Bonferroni correction to determine our significance threshold, which divides the nominal significance level of 0.05 by the number of tests performed. Thus, our p-value threshold for declaring significance was 0.05/9 = 0.0056. As detailed below, two of the nine features were found to be significantly non-random by this criterion and seven were found to not be significant:

### Significant Features

#### Significant Feature #1

Localization to the integrin (cell) binding site: p-value  = 0.0024

Details: Three of eleven peptides (two unique sequences) were observed to overlap with the integrin binding site of the fibril which we define as comprising residues 502–510.

#### Significant Feature #2

Co-localization between the two species: p-value  = 0.0034

Details: Three pairs of peptides (three unique sequences) from the two species co-localized on the collagen monomer.

### Non-Significant Features

#### Non-Significant Feature #1

Overlap zone vs. gap zone: p-value  = 0.022

Details: Ten of eleven peptides (seven unique sequences) localized to the overlap zone.

#### Non-Significant Feature #2

Cell interaction domain: p-value  = 0.212

Details: Three of eleven peptides (two unique sequences) localized to the cell interaction domain.

#### Non-Significant Feature #3

Monomers 2, 3, and 4: p-value  = 0.016

Details: All peptides (eight unique sequences) mapped to monomers 2, 3, and 4, and none to monomers 1 and 5.

#### Non-Significant Feature #4

Co-localization of peptides: p-value  = 0.036

Details: Four of the eleven peptides (four unique sequences) did not overlap with any other peptides.

#### Non-Significant Feature #5

Overlap with cross-links: p-value  = 0.097

Details: Five of the eleven peptides (three unique sequences) overlapped with the intermolecular cross-links.

#### Non-Significant Feature #6

Overlap with any functional domain: p-value  = 0.014

Details: Eight out of eleven peptides (five unique sequences) co-localized with at least one of the following functional domains: the central integrin binding site; MMP-1-cleavage site; decoron ligation sequences; and overlapping of the intermolecular crosslinks, or aligning with them across the fibril.

#### Non-Significant Feature #7

Overlap with the master control region: p-value  = 0.018

Details: Ten of eleven peptides (seven unique sequences) occupied the master control region, a fibril zone where most of the collagen fibrils crucial functional sequences are located.

## References

[1] Nielsen-Marsh CM (2002). Biomolecules in fossil remains..

[2] Buckley M, Walker A, Ho SYW, Yang Y, Smith C (2008). Comment on 'Protein sequences from mastodon and Tyrannosaurus rex revealed by mass spectrometry.. Science.

[3] Pevzner PA, Kim S, Ng J (2008). Comment on “Protein sequences from mastodon and Tyrannosaurus rex revealed by mass spectrometry”.. Science.

[4] Schweitzer MH, Wittmeyer JL, Horner JR (2007a). Soft tissue and cellular preservation in vertebrate skeletal elements from the Cretaceous to the present.. Proc R Soc Lond B.

[5] Asara JM, Schweitzer MH, Phillips MP, Freimark LM, Cantley LC (2007a). Protein sequences from mastodon (Mammut americanum) and dinosaur (Tyrannosaurus rex) revealed by mass spectrometry.. Science.

[6] Schweitzer MH, Suo Z, Avci R, Asara JM, Allen MA (2007b). Analyses of soft tissue from Tyrannosaurus rex suggest the presence of protein.. Science.

[7] Asara JM, Garavelli JS, Slatter DA, Schweitzer MH, Freimark LM (2007b). Interpreting sequences from mastodon and Tyrannosaurus rex.. Science.

[8] Organ CL, Schweitzer MH, Zheng W, Freimark LM, Cantley LC (2008). Molecular phylogenetics of mastodon and Tyrannosaurus rex.. Science.

[9] Schweitzer MH, Zheng W, Organ CL, Avci R, Suo Z (2009). Biomolecular characterization and protein sequences of the Campanian hadrosaur Brachylophosaurus canadensis.. Science.

[10] Lindahl T (1993). Instability and decay of the primary structure of DNA.. Nature.

[11] Hoss M (2000). Nanderthal population genetics.. Nature.

[12] Qian YR, Engel MH, Macko SA, Carpenter S, Deming J (1993). Kinetics of peptide hydrolysis and amino acid decomposition at high temperature.. Geochim Cosmochim Acta.

[13] Bada JL, Wang XS, Hamilton H (1999). Preservation of key biomolecules in the fossil record: current knowledge and future challenges.. Phil Trans R Soc Lond B.

[14] Collins MJ, Riley M, Child AM, Turner-Walker G (1995). A basic mathematical simulation of the chemical degradation of ancient collagen.. J Archaeol Sci.

[15] Lindqvist C, Schuster SC, Sun Y, Talbot SL, Qi J (2010). Complete mitochondrial genome of a Pleistocene jawbone unveils the origin of polar bear.. Proc Natl Acad Sci USA.

[16] Collins MJ, Gernaey A, Nielsen-Marsh CM, Vermeer C, Westbroek P (2000). slow rates of degradation of osteocalcin: green light for fossil bone protein?. Geology.

[17] Tuross N (1989). Albumin preservation in the Taima-taima mastodon skeleton.. Appllied Geochemistry.

[18] Salamon M, Tuross N, Arensburg B, Weiner S (2005). Relatively well preserved DNA is present in the crystal aggregates of fossil bones.. Proc Natl Acad Sci USA.

[19] Kaye TG, Gaugler G, Sawlowicz Z (2008). Dinosaurian soft tissues interpreted as bacterial biofilms.. PLoS ONE.

[20] Tuross N (2002). Alterations in fossil collagen.. Archaeometry.

[21] Notbohm H, Mosler S, Bodo M, Yang C, Lehmann H (1992). Comparative study on the thermostability of collagen I of skin and bone: Influence of posttranslational hydroxylation of prolyl and lysyl residues.. Journal of Protein Chemistry.

[22] Hanson DA, Eyre DR (1996). Molecular site specificity of pyridinoline and pyrrole cross links in Type I collagen of human bone.. Journal of Biological Chemistry.

[23] Nemethy G, Scheraga HA (1986). Stabilization of collagen fibrils by hydroxyproline.. Biochemistry.

[24] Miles CA, Ghelashvili M (1999). Polymer-in-a-box mechanism for the thermal stabilization of collagen molecules in fibers.. Biophysical Journal.

[25] Wojtowicz A, Yamauchi M, Montella A, Bandiera P, Sotowski R (1999). Persistence of bone collagen cross-links in skeletons of the Nuraghi population living in Sardinia 1500-1200 B. C. Calcified Tissue International.

[26] Collins MJ, Nielsen-Marsh CM, Hiller J, Smith CI, Roberts JP (2002). The survival of organic matter in bone: a review.. Archaeometry.

[27] Sykes GA, Collins MJ, Walton DI (1995). The significance of a geochemically isolated intracrystalline organic fraction within biominerals.. Organic Geochemistry.

[28] Trueman CN, Martill DM (2002). The long-term survival of bone: the role of bioerosion.. Archaeometry.

[29] Schmidt-Schultz TH, Schultz M (2004). Bone protects proteins over thousands of years: extraction, analysis, and interpretation of extracellular matrix proteins in archeological skeletal remains.. American Journal of Physical Antrhopology.

[30] Salmon V, Derenne s, Lallier-Verges E, Largeau C, Beaudoin B (2000). Protection of organic matter by mineral matrix in a Cenomanian black shale.. Organic Geochemistry.

[31] Van der Rest M, Garrone R (1991). Collagen family of proteins.. FASEB Journal.

[32] Weiner S, Traub W, Wagner HD (1999). Lamellar bone: Structure-function relations.. Journal of Structural Biology.

[33] Sweeney SM, Orgel JP, Fertala A, McAuliffe JD, Turner KR (2008). Candidate cell and matrix interaction domains on the collagen fibril, the predominant protein of vertebrates.. Journal of Biological Chemistry.

[34] Perumal S, Antipova O, Orgel JP (2008). Collagen fibril architecture, domain organization, and triple-helical conformation govern its proteolysis.. Proc Natl Acad Sci USA.

[35] Orgel JP, Irving TC, Miller A, Wess TJ (2006). Microfibrillar structure of type I collagen in situ.. Proc Natl Acad Sci USA.

[36] Orgel JP, Wess TJ, Miller A (2000). Structure.

[37] Bern M, Phinney BS, Goldberg D (2009). Reanalysis of Tyrannosaurus rex mass spectra..

[38] Miller EJ, Reddi AH, Piez KA (1984). Chemistry of the collagens and their distribution;.

[39] Hu XW, Knight DP, Chapman JA (1997). The effect of non-polar liquids and non-ionic detergents on the ultrastructure and assembly of rat tail tendon collagen fibrils in vitro.. Biochimica Biophysica Acta.

[40] Miles CA, Bailey AJ (2001). Thermally labile domains in the collagen molecule.. Micron.

[41] Buckley M, Collins MJ, Thomas-Oates J, Wilson JC (2009). Species identification by analysis of bone collage using matrix-assisted laser desorption/ionisation time-of-flight mass spectrometry.. Rapid communications in mass spectrometry.

[42] Buckley M, Larkin N, Collins MJ (2011). Mammoth and mastodon collagen sequences: survival and utility.. Geochim Cosmochim Acta.

[43] Schweitzer MH, Wittmeyer JL, Horner JH, Toporski JB (2005). Soft Tissue Vessels and Cellular Preservation in Tyrannosaurus rex.. Science.

[44] Chapman JA (1974). The staining pattern of collagen fibrils: I. an analysis of electron micrographs.. Connective Tissue Research.

[45] Ostrom P, Gandhi H, Strahler J, Walker A, Andrews P (2006). Unraveling the sequence and structure of the protein osteocalcin from a 42 ka fossil horse.. Geochim Cosmochim Acta.

[46] A. CJ, R. S, T.O. B (1996). Computational Chemistry.

